# EXPLORING TYPE I INTERFERON SIGNALING AS A TRANSLATIONAL TARGET IN PVT1 EXON 9 OVEREXPRESSED NEUROENDOCRINE PROSTATE CANCER

**DOI:** 10.51894/001c.122802

**Published:** 2024-08-30

**Authors:** Rachel Bonacci, Meghan McGill, Colin Finnegan, Chinedum Udekwu, Baris Boylu, Olorunseun O. Ogunwobi

**Affiliations:** 1 College of Osteopathic Medicine Michigan State University

## 05

### INTRODUCTION/BACKGROUND

Plasmacytoma variant translocation 1 (PVT1) is an oncogene overexpressed in prostate cancer (PCa) that can alter gene expression directly and can act as a noncoding RNA sponge. Our group previously identified overexpression of PVT1 exon 9 in PCa samples derived from men of African Ancestry and overexpression may somewhat explain why this group experiences more aggressive disease and worse overall outcomes. To explore the pathogenicity of PVT1 exon 9, we overexpressed in RWPE1 epithelial prostate cells (RWPE1_ex9) and observed enhanced migration and invasion compared to empty vector (RWPE1_EV). Injection of RWPE1_ex9 cells in vivo led to neuroendocrine small-cell PCa (NEPC) development that was pathologically confirmed. Based on these results, we concluded PVT1 exon 9 is pathogenic in prostate cancer.

### OBJECTIVES/HYPOTHESIS

We wanted to explore the downstream molecular targets of PVT1 exon 9 from RNA-sequencing data to identify actionable therapeutic targets and experimentally validate candidate targets in vitro.

### METHODS

We utilized genetic and biochemical techniques to validate PVT1 exon 9 targets.

### RESULTS

RNA-sequencing analysis of RWPE1_ex9 revealed significant (p-value < 0.05) upregulation of 141 genes including the Type I interferon signaling genes RSAD2 and CMPK2, compared to RWPE1_EV. Within a paired prostate model we previously generated from 22RV1 castrate-resistant (CRPC) cells injected in vivo, we found overexpression of PVT1 exon 9, RSAD2 and CMPK2 within circulating tumor cells (C22OH) compared to primary tumor cells (T22OH) and parental 22RV1 model. Compared to primary tumor, circulating tumor cells expressed the neuroendocrine marker chromogramin A and lacked androgen receptor (AR) signaling suggestive of a transformation towards NEPC. In both RWPE1_ex9 and C22OH we observed significant expression of interferon-gamma. Knockdown of RSAD2 within the C22OH model led to ~50% decreased cell viability as well as re-expression of AR. These results are clinically relevant as patient expression data suggests RSAD2 is upregulated in both CRPC and NEPC subtypes compared to benign prostate tissue and hormone sensitive prostate cancer.

### DISCUSSION/CONCLUSIONS

Based on our results, we hypothesize RSAD2 is a promising translational target either alone or in conjunction with AR or interferon-gamma inhibition and may be a useful strategy to target NEPC.

